# Biological Control for Grapevine Crown Gall Evaluated by a Network Meta-Analysis

**DOI:** 10.3390/plants12030572

**Published:** 2023-01-27

**Authors:** Akira Kawaguchi, Namiko Kirino, Koji Inoue

**Affiliations:** 1 Western Region Agricultural Research Center (WARC) (Kinki, Chugoku and Shikoku Regions), National Agriculture and Food Research Organization (NARO), 6-12-1 Nishifukatsu-cho, Fukuyama 721-8514, Hiroshima, Japan; 2 Research Institute for Agriculture, Okayama Prefectural Technology Center for Agriculture, Forestry and Fisheries, 1174-1 Koudaoki, Akaiwa City 709-0801, Okayama, Japan

**Keywords:** grapevine crown gall, biological control, field trial, network meta-analysis, *Allorhizobium vitis*

## Abstract

Grapevine crown gall (GCG), which is caused by *Allorhizobium vitis* (=*Rhizobium vitis*) tumorigenic strains, is the most important disease of grapevine around the world. Previously, nonpathogenic *A. vitis* strains VAR03-1, ARK-1, ARK-2, and ARK-3 were identified as promising biological control agents, but the control effects of each strain were not directly compared and assessed in the field because field trials were conducted in different fields and years. Thus, the results of the control effects obtained from 16 field trials in 12 years from 2006 to 2017 were analyzed and evaluated by a linear mixed model (LMM) and a network meta-analysis (NMA). The results of the LMM strongly indicate that the factor “antagonistic strain” was significantly related to the biological control activity in this study, but the other factors, “concentration of cell suspension”, “field”, and “year”, were not. Then, the results of 16 field trials were combined in an NMA. The estimated relative risk (RR) after treatment with ARK-1, ARK-2, ARK-3, VAR03-1, and K84 were 0.16, 0.20, 0.22, 0.24, and 0.74, respectively. In conclusion, strain ARK-1 was the best antagonist regardless of the concentration of the cell suspension, field, and year differences, and it can be recommended to control GCG.

## 1. Introduction

Grapevine (*Vitis vinifera* L.) crown gall (GCG) is caused mainly by *Allorhizobium vitis* (Ti) (syn. *Rhizobium vitis* (Ti), *Agrobacterium vitis* (Ti), and *A. tumefaciens* biovar 3), where Ti means tumorigenic [1,2]. *A. vitis* (Ti) infects grapevines through wounds, such as freezing injuries, cutting damage, and grafting [3,4,5,6,7,8]. GCG is a pandemic throughout the world [3,4,5,6]. Galls generally form on the trunks and cordons of young and mature grapevines [3,4,5,6,7,8,9]. Infected grapevines often experience inferior growth, but the galls cause grapevine death [7].

The most serious problem is that there is no effective and practical method to manage GCG. Some chemical control measures, which are copper bactericides and antibiotics, are able to kill the bacterium upon contact, but they do not penetrate the grapevines and contact with Ti strains residing inside systemically [10]. *Rhizobium rhizogenes* (=*A. rhizogenes* and *A. radiobacter* biovar 2) strain K84 suppresses gall incidence caused by Ti strains of *R. rhizogenes* [11,12,13,14,15], but K84 is not effective against GCG, which is caused by *A. vitis* (Ti) [3,6,16,17]. Previously, we reported that the nonpathogenic and antagonistic *A. vitis* strains VAR03-1 and ARK-1 inhibited gall formation in not only grapevine but in diverse plant species [5,6,7,10,16,17,18,19,20,21,22,23,24,25,26,27,28,29,30,31,32,33,34]. In particular, strain ARK-1 strongly controlled GCG in vineyards by several unique biological control mechanisms. ARK-1 suppressed the population growth of Ti strains in grapevines [26,27], migrated inside grapevines [33], suppressed the expression of virulence (*vir*) genes [6,27,30], and primed the induction of the *LOX-9* gene, which was one of the defense genes of grapevine used as a marker of jasmonic acid (JA) signaling [34].

The final purpose of this study was to utilize these antagonistic strains as a new biopesticide. We previously evaluated the control effects of ARK-1 and VAR03-1 against GCG in several field trials by a meta-analysis (MA), which is a statistical technique for combining the findings from multiple studies [21,25,35]. In a conventional pairwise meta-analysis, researchers collect experiments or studies that evaluate the same treatment, create pairs of treatment and control groups, and directly compute the effect size (direct treatment comparison) [36]. However, the effects of ARK-1 and VAR03-1 strains could not be compared directly because those field trials were conducted separately in different field locations and years. In addition, there is no evidence of the effectiveness of treatment with strains ARK-2 and ARK-3 in controlling GCG in the field. Recently, network meta-analysis (NMA) has been used to combine evidence on multiple studies comparing multiple treatments [36]. NMA allows to combine direct and indirect evidence. For example, the comparison of treatments X and Y is performed using both studies that directly compare X with Y (direct evidence) and studies that compare X with Z and Y with Z (indirect evidence) [36]. Therefore, the objective of this study was to assess the control effects of ARK-1, ARK-2, ARK-3, and VAR03-1 against GCG in different 16 fields trials over 12 years by carrying out an NMA.

## 2. Results

### 2.1. Regression Analysis by Linear Mixed Model (LMM)

An LMM of the risk ratio (RR) values after treatments with ARK-1, ARK-2, ARK-3, VAR03-1, and K84 compared with water treatment as an objective variable showed that the significant (*p* ≤ 0.05) explanatory variable was “antagonistic strain” alone (*p* = 0.0005), suggesting that other factors, such as “cell concentration”, “field”, and “year”, were not significantly related to the biological control activity in this study (Table 1).

### 2.2. Biological Control Effects Combined by Network Meta-Analysis (NMA)

**Figure 1:**
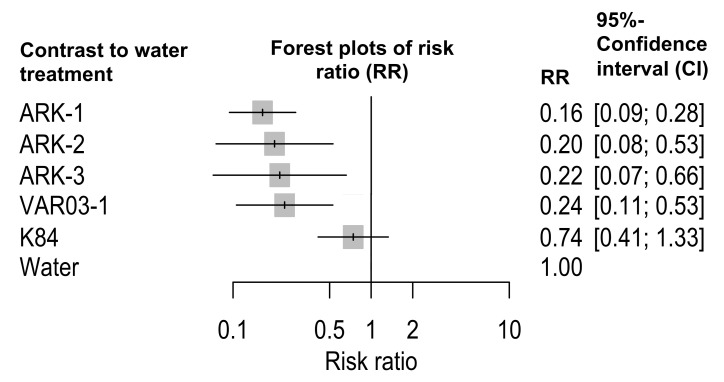
Evaluation based on a network meta-analysis (NMA) of the effects of the nonpathogenic *Allorhizobium vitis* strains ARK-1, ARK-2, ARK-3, and VAR03-1 and *Rhizobium rhizogenes* strain K84 on grapevine crown gall (GCG) in 16 different field experiments. In the forest plots, each gray square marks the value of the risk ratio compared with the water treatment. The spread (horizontal line) indicates the 95% confidence interval.

An NMA of 16 field trials performed over 12 years from 2006 to 2017 of the biological control effects of ARK-1, ARK-2, ARK-3, VAR03-1, and K84 treatments on the GCG compared with the water treatment showed that the total estimated RRs after treatments with ARK-1, ARK-2, ARK-3, VAR03-1, and K84 compared with water treatment were 0.16 (95% confidence interval (CI): 0.09–0.28, *p* < 0.0001), 0.20 (95% CI: 0.08–0.53, *p* = 0.0012), 0.22 (95% CI: 0.07–0.66, *p* = 0.0071), 0.24 (95% CI: 0.11–0.53, *p* = 0.0004), and 0.74 (95% CI: 0.41–1.33, *p* = 0.3162), respectively (Figure 1).

**Figure 2:**
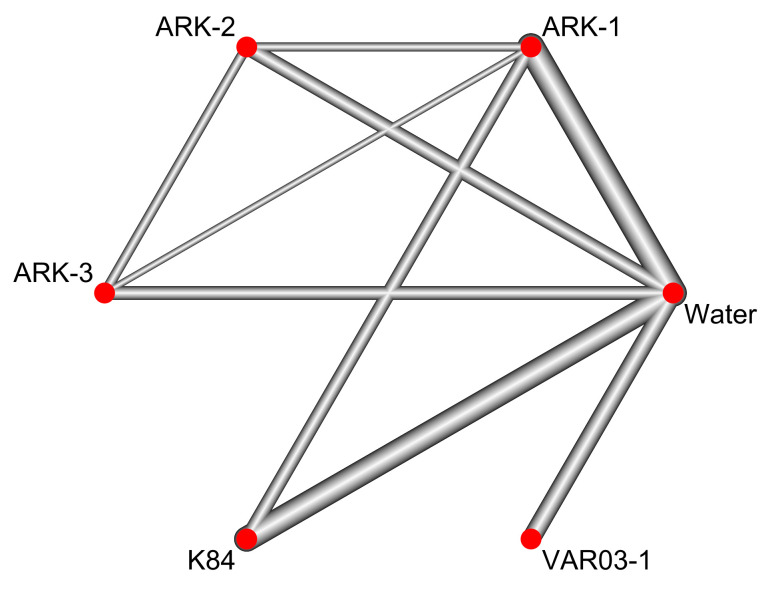
A network map of the meta-analyses (NMA). The connected lines show direct comparisons, and the unconnected lines show indirect comparisons. A wider line shows a larger number of field trials.

In 16 field trials, the ARK-3, K84, and VAR03-1 treatments were indirectly compared with each other (Figure 2). The ARK-2, K84, and VAR03-1 treatments were also indirectly compared with each other (Figure 2). The VAR03-1 treatment was directly compared with the water treatment alone (Figure 2). Both the *I^2^* and *τ*^2^ values were zero. In addition, the results of a total *Q* test, heterogeneity *Q* test (within designs), and inconsistency *Q* test (between designs) were *p* = 0.9988, *p* = 0.9954, and *p* = 0.9077, respectively (Table 2), indicating the absence of heterogeneity and inconsistency within and between study designs. Thus, it seemed that the result of the NMA was reasonable.

## 3. Discussion

In the co-inoculation test with a 1:1 cell ratio of pathogen/nonpathogen into stems of tomato and grapevine showed that the gall inhibition activity of ARK-1 tended to be higher than that of the other strains, including ARK-2, ARK-3, K84, and VAR03-1, tested in the greenhouse experiments [17]. However, even if good results were produced in the laboratory and greenhouse experiments, the field trials were not always successful. In this study, the results of the treatments with ARK-1, ARK-2, ARK-3, and VAR03-1 indicated that the disease incidence was significantly reduced gall incidence (Figure 1). The *R. rhizogenes* strain K84 did not significantly reduce the GCG incidence, again demonstrating that K84 does not control GCG caused by *A. vitis* (Ti) in the field [3,6,16,17]. Especially, the RR value of the ARK-1 treatment was the lowest, and the range of the 95% CI was the smallest (Figure 1). The RR value of 0.16 indicates that the GCG incidence during the ARK-1 treatment decreased to 16% of that of the water treatment and that the control effect was extremely high in the field. In this study, ARK-1 was the best antagonistic strain, and it can be recommended to control GCG.

In the results of the LMM, the concentration of a cell suspension of an antagonistic strain, tested field, and year were not significantly related to RR as biocontrol activity (Table 1), indicating that these antagonists, except strain K84, might stably control GCG regardless of fields and years. Strain K84 does not prevent the initial infection of grapevine by *A. vitis* Ti strains, because *A. vitis* Ti strains are insensitive to agrocin 84 produced by K84 [3,5,6]. In this study, all field trials were conducted in different fields but in the same location and were under the same weather conditions in each year. Thus, “year” as an explanatory variable could include the effect of the weather conditions. However, the “year” factor was not significantly related to the biological control activity (Table 1), indicating that the weather conditions in each year might not be significant either. The grapevine roots were soaked in a cell suspension of an antagonist for one hour and planted. ARK-1 can colonize inside roots, move inside plant stems, rapidly suppress the virulence related-genes of *A. vitis* (Ti) strain, and prime the induction of certain defense genes in plants [25,27,30,33,34]. It seems that this treatment method is less susceptible to soil and other environmental conditions. The concentration of the cell suspension of antagonistic microorganisms is important for biological control, and it is thought that a higher concentration is better for the control effect in general [25]. In practical use, however, the lower concentration of a cell suspension contributes to the lower cost of plant disease control for farmers. In this study, the concentration of the cell suspensions of the antagonistic strains was not significantly related to the RR (Table 1), and the ARK-1 treatments in the four field trials from 2013 to 2017 were carried out using the lowest cell suspension (5.0 × 10^7^ cells/mL) (Table 3). This result shows that the cell suspension of 5.0 × 10^7^ cells/mL ARK-1 might be suitable for practical use.

Field trials are an essential part of the development of new technology for agriculture and especially important in developing a biological control procedure. Even though positive results may be produced in laboratory and greenhouse experiments, field trials often do not show the expected results. We carried out some field trials [21,25], but there were sometimes constraints in conducting them. For example, if many treatments, plots, and replications are set in one experimental field, the sample size becomes very small, or the soil condition is worse during an experimental period due to the unexpected heavy rain, and data may not be obtained in some plots. However, an NMA can compute the effect size between treatment groups, including some indirect treatments, even if there is no direct comparison [35]. In this study, using an NMA, the control effects of some antagonistic strains obtained in 16 field trials conducted in different fields and years (Table 3) were estimated. 

Several laboratories have attempted to identify other biocontrol agents for GCG [37,38,39,40,41,42,43,44,45,46]. The *A. vitis* strain F2/5 suppressed the growth of *A. vitis* (Ti) strains on medium plates and inhibited GCG in stem-wounding experiments in greenhouse trials [37,38,39,40,41]. Wang et al. [44] reported that an antibacterial compound named “Ar26” produced by *A. vitis* strain E26 suppressed the growth of *A. vitis* (Ti) on medium plates. Chen et al. [43] reported that *Rahnella aquatilis* strain HX2 showed an inhibitory effect on the development of GCG. As described above, several researchers have tried to progress other biological control agents for GCG and reported potential bacterial and fungal strains, but they did not show a positive effect of these candidate antagonistic strains in field trials and have not produced a successful candidate until now. In contrast, we have shown only the good results of biological control for GCG in 16 field trials over 12 years in this study and our previous reports [5,6,21,22,24,25,26]. We are now developing a new bactericide made from ARK-1 and obtaining the positive result that the new ARK-1 bactericide treatment is effective in controlling GCG and other plant species in field trials. We will achieve the development of the new biopesticide in the near future.

## 4. Materials and Methods

### 4.1. Field Trials of the biological Control for Grapevine Crown Gall (GCG)

The details of all 16 field trials (2006-A, 2007-A, 2007-B, 2009-A, 2009-B, 2009-C, 2010-A, 2010-B, 2011-A, 2011-B, 2012-C, 2013-A, 2015-A, 2016-D, and 2017-D) are described in Table 3. These trials of the biological control of GCG were designed as randomized or systematic controlled trials and carried out in four different experimental fields, A (2006, 2007, 2009, 2010, 2011, 2012, 2013, and 2015), B (2009, 2010, and 2011), C (2007 and 2012), and D (2016 and 2017) in Akaiwa City, Okayama, Japan. Trials 2006-A, 2007-A, 2007-B, 2009-A, 2009-C, 2010-A, 2011-A, 2011-B, and 2012-C were previously reported [21,25]. One month before a trial, a commercial organic fertilizer (Temporon, containing N = 0.77%, P = 0.09%, K = 0.08%, lignocellulose, humic acid, B, Mg, Ca, and Mn; Mitsubishi-Shoji, Tokyo, Japan) was applied at a rate of 4.0–5.0 kg/m^2^ and thoroughly incorporated into the soil every year. All fields were contaminated by *A. vitis* (Ti) strains [25]. Two weeks before each trial, 20 L/m^2^ of a mixed cell suspension (approximately 10^8^ cells/mL) of several *A. vitis* (Ti) strains, which were isolated from various vineyards and areas in Japan, was poured onto the soil [25]. Nonpathogenic *A. vitis* strains ARK-1, ARK-2, ARK-3, and VAR03-1 were used from stocks preserved at −80 °C, and the commercial nonpathogenic *R. rhizogenes* strain K84 (Bacterose, Nihon Noyaku, Tokyo, Japan) was used. Cell suspensions of strains ARK-1, ARK-2, ARK-3, VAR03-1, and K84 were prepared from 48 h slant cultures grown on PS medium and adjusted to OD600 = 0.05–1.0 (corresponding to approximately 5.0 × 10^7^ cells/mL–1.0 × 10^9^ cells/mL). In each field trial, the concentrations of the cell suspension were different (Table 3). The roots of grapevines were pruned to half and soaked for 1 h in a cell suspension of each strain or water, and then those plants were planted in each plot. Gall formation on roots and stems of grapevines was investigated after 6 to 10 months. The rainy season in Japan is from June to July. The temperature ranged from 12 to 37 °C, and no severe damage due to the fact of insects and weather conditions was observed during cultivation.

### 4.2. Regression Analysis

To clarify the factors affecting the biological control of antagonistic strains in 16 field trials, a regression analysis based on a linear mixed model (LMM) was performed. In this study, we followed the experimental methods described in previous reports [9,47]. The parameters, which were an antagonistic strain (categorical numbers: 0 = ARK-1, 1 = ARK-2, 2 = ARK-3, 3 = VAR03-1, an d4 = K84), concentrations of cell suspensions of the antagonist (categorical numbers: 0 = 5.0 × 10^7^ cells/mL, 1 = 1.0 × 10^8^ cells/mL, 2 = 2.0 × 10^8^ cells/mL, and 3 = 1.0 × 10^9^ cells/mL), field (categorical numbers: 0 = field A, 1 = field B, 2 = field C, and 3 = field D), and year (categorical numbers: 0 = 2006, 1 = 2007, 2 = 2008, 3 = 2009, 4 = 2010, 5 = 2011, 6 = 2012, 7 = 2013, 8 = 2015, 9 = 2016, and 10 = 2017) were coded and defined as explanatory variables. Individual field trials (categorical numbers from 1 to 16) were defined as the y-intercepts of random effects. The objective variable was the value of the risk ratio (RR) of each antagonist’s treatment, which was defined in this study as RR = (proportion of plants developing galls with the antagonist treatment) / (proportion of plants developing galls with water treatment). The R (ver. 3.6.1, R Development Core Team) package “lme4” was used to estimate regression coefficients.

### 4.3. Network Meta-Analysis (NMA)

The disease incidences in the 16 different field trials of ARK-1, ARK-2, ARK-3, K84, and VAT03-1 treatments were subjected to NMA using a random effect model. Before performing NMA, all data of field trials (Table 3) were pre-treated using a “pairwise” function, which can transform data with continuous, binary, or generic outcomes as well as incidence rates from an arm-based to a contrast-based format, by the R package “netmeta” [48]. After it is transformed, an NMA based on frequentist method was performed using the R package “netmeta” [48].

The effect size of antagonistic treatment was calculated as total estimated RR [48]. In evaluating the control effect, a low RR indicated a high control effect. To test for heterogenicity and inconsistency of NMA results, Cochran’s *Q* test, *I^2^*, and *τ*^2^ values were calculated by the R package “netmeta” [48].

## 5. Conclusions

An NMA of 16 field trials over 12 years comparing the effectiveness of ARK-1, ARK-2, ARK-3, VAR03-1, and K84 suggested the superiority of ARK-1 to all other tested strains and revealed strong evidence that ARK-1 was effective to manage GCG by application in the field. We hope that a new bactericide made from ARK-1 based on our studies will contribute to managing GCG in agriculture around the world.

## Figures and Tables

**Table 1:** Parameter estimates for the best-fit linear mixed model (LMM) for the factors related with the risk ratio (RR) of the biological control effect for grapevine crown gall (GCG) in 16 field trials over 12 years.

Objective Variable	Explanatory Variable	Parameter Estimate	Standard Error	*t*-Value	*p*-Value
Risk ratio (RR)	*y*-Intercept ^a^	0.460	0.436	1.049	0.3297
	Antagonistic strain	0.124	0.027	4.697	0.0005
	Concentration of cell suspension	−0.174	0.142	−1.225	0.2473
	Field	−0.005	0.054	−0.095	0.9256
	Year	−0.037	0.058	−0.634	0.5409

^a^ Standard error of each experiment, which was defined as the y-intercept of the random effects, was estimated as 0.018.

**Table 2:** The Cochran’s Q test of heterogeneity (within designs) and inconsistency (between designs).

Tests	*Q*	df	*p*-Value
Total	4.51	17	0.9988
Within designs	3.50	13	0.9954
Between designs	1.01	4	0.9077

**Table 3:** Details of the 16 field trials.

Experiment (Year-Field ID) ^a^	Events in Treatment 1		Events in Treatment 2		Events in Treatment 3		Events in Treatment 4		Treatment 1	Treatment 2	Treatment 3	Treatment 4	Concentration of Each Strain (cells/mL)	Grapevine Nursery Stock (2 Year Olds) ^b^		Plot Arrangement	Plot Size (m)	No. of Rows/Plots	No. of Plots/Treatments	No. of Plants/Plots	The Date of Planted/Investigated	Reference
	Galled Plants	Total Plants	Galled Plants	Total Plants	Galled Plants	Total Plants	Galled Plants	Total Plants						Scion Cultivar	Rootstock							
2006-A	4	30	11	30	-	-	-	-	VAR03-1	Water	-	-	1 × 10^9^	Pione	Teleki-Kober 5BB	Systematic	8.0 × 3.0	3 rows spaced 50 cm apart and 100 cm between plants	2	15	28 March/28 September	[25]
2007-A	0	42	6	42	-	-	-	-	VAR03-1	Water	-	-	1 × 10^9^	Pione	Teleki-Kober 5BB	Randomized	8.0 × 3.0	2 rows spaced 60 cm apart and 40 cm between plants	3	14	19 April/27 November	[21,25]
2007-B	1	45	13	45	-	-	-	-	VAR03-1	Water	-	-	1 × 10^9^	Neo Muscat	Own-root	Randomized	1.6 × 1.5	6 rows spaced 15 cm apart and 15 cm between plants	3	15	13 Febru-ary/12 October	[21,25]
2009-C	1	24	4	24	-	-	-	-	VAR03-1	Water	-	-	1 × 10^9^	Pione	Teleki-Kober 5BB	Randomized	7.0 × 1.0	1 row spaced 50 cm between plants	3	8	21 April/4 November	[25]
2009-A	1	30	8	30	-	-	-	-	ARK-1	Water	-	-	2 × 10^8^	Pione	Teleki-Kober 5BB	Randomized	6.0 × 1.0	1 row spaced 50 cm between plants	3	10	11 May/4 November	[25]
2009-B	1	24	3	24	3	24	3	24	ARK-1	Water	ARK-2	ARK-3	2 × 10^8^	Pione	Teleki-Kober 5BB	Randomized	1.6 × 1.5	1 row spaced 40 cm between plants	6	4	25 April/9 January	This study
2010-A	0	16	4	16	-	-	-	-	ARK-1	Water	-	-	2 × 10^8^	Pione	Teleki-Kober 5BB	Systematic	6.0 × 1.0	1 row spaced 40 cm between plants	2	8	26 May/5 October	[25]
2010-B	1	36	7	36	1	36	-	-	ARK-1	Water	ARK-2	-	1 × 10^8^	Pione	Teleki-Kober 5BB	Randomized	1.6 × 1.5	2 rows spaced 60 cm apart and 40 cm between plants	6	6	10 March/18 October	This study
2011-A	2	20	9	20	-	-	-	-	ARK-1	Water	-	-	1 × 10^8^	Pione	Teleki-Kober 5BB	Systematic	6.0 × 1.0	1 row spaced 50 cm between plants	2	10	28 March/5 December	[25]
2011-B	2	40	14	40	-	-	-	-	ARK-1	Water	-	-	1 × 10^8^	Pione	Teleki-Kober 5BB	Randomized	1.6 × 1.5	2 rows spaced 60 cm apart and 30 cm between plants	4	10	24 March/21 December	[25]
2012-A	0	38	2	40	2	39	-	-	ARK-1	Water	K84	-	1 × 10^8^	Pione	Teleki-Kober 5BB	Randomized	6.0 × 1.0	1 row spaced 60 cm between plants	3	10	5 April/6 November	This study
2012-C	1	29	4	27	-	-	-	-	ARK-1	Water	-	-	1 × 10^8^	Pione	Teleki-Kober 5BB	Randomized	7.0 × 1.0	1 row spaced 50 cm between plants	3	10	10 April/27 October	[25]
2013-A	3	48	14	48	11	48	-	-	ARK-1	Water	K84	-	5 × 10^7^	Pione	Teleki-Kober 5BB	Randomized	5.0 × 0.8	1 row spaced 30 cm between plants	3	16	5 April/15 October	This study
2015-A	0	28	3	30	1	29	-	-	ARK-1	Water	K84	-	5 × 10^7^	Pione	Teleki-Kober 5BB	Randomized	5.0 × 0.8	1 row spaced 30 cm between plants	3	10	8 April/20 October	This study
2016-D	0	28	4	30	-	-	-	-	ARK-1	Water	-	-	5 × 10^7^	Pione	Teleki-Kober 5BB	Randomized	4.0 × 0.8	1 row spaced 40 cm between plants	3	10	8 April/10 October	This study
2017-D	0	10	2	10	-	-	-	-	ARK-1	Water	-	-	5 × 10^7^	Kyoho	Teleki-Kober 5BB	Randomized	4.0 × 0.8	1 row spaced 40 cm between plants	1	10	8 April/22 October	This study

^a^ Same letter (A, B, C and D) shows the same experimental field. ^b^ *Vitis vinifera* × *V. labrusca* cv. Pione, *V. vinifera* cv. Neo Muscat, *V. labrusca* × *V. vinifera* cv. Kyoho, and *V. cinerea* var. *helleri* × *V. riparia* cv. Teleki-Kober 5BB.

## Data Availability

The data presented in this study are available upon request from the corresponding author.
